# Kasai-Like Hepaticojejunostomy As Salvage Reconstruction for Bile Duct Injury in an Infant: A Case Report and Technical Consideration

**DOI:** 10.7759/cureus.103176

**Published:** 2026-02-07

**Authors:** Masaya Yamoto, Shohei Kishida, Marie Todo, Yuichi Takama, Masafumi Kamiyama

**Affiliations:** 1 Department of Pediatric Surgery, Osaka City General Hospital, Osaka, JPN

**Keywords:** bile duct injury, hepaticojejunostomy, hepatoblastoma, jejunal patch, kasai procedure, pediatric hepatectomy, salvage reconstruction

## Abstract

Bile duct injury (BDI) in children is an uncommon yet serious complication after hepatobiliary surgery or trauma. Management is particularly challenging because of the extremely small caliber of infantile bile ducts, which makes conventional biliary reconstruction technically difficult. Standard approaches are often not feasible when the injury occurs at the hilar level or involves ultra-thin ducts. In such cases, alternative surgical strategies are required. A Kasai-like hepaticojejunostomy- an approach analogous to the portoenterostomy used in biliary atresia- may serve as a salvage option when standard hepaticojejunostomy is technically impossible. We report a one-year-old boy with multiple congenital anomalies and hepatoblastoma involving segments 4 and 6 who underwent right hepatic trisectionectomy. During hilar dissection, an inadvertent pin-hole injury to the common bile duct (<1 mm diameter) was encountered, precluding standard reconstruction. A Kasai-type salvage was performed: the bile duct incision was extended proximally to the left duct, a 3-Fr transanastomotic stent was inserted, and a Roux-en-Y jejunal limb was directly anastomosed to the transected hepatic surface. Postoperative recovery was uneventful, with no bile leakage. The stent was removed three months later, and pathology confirmed complete resection of hepatoblastoma. This case demonstrates the feasibility of Kasai-like hepaticojejunostomy as a selective salvage procedure for iatrogenic hilar bile duct injury in infants. While the successful outcome in this single case is encouraging, it is important to recognize that this approach was necessitated by extreme anatomical constraints that precluded conventional reconstruction. When duct size makes standard hepaticojejunostomy technically impossible, this technique offers a safe alternative; however, further evaluation is needed to establish its long-term role compared to standard methods.

## Introduction

Bile duct injury (BDI) during complex liver tumor resection, especially involving the hepatic hilum, remains a daunting technical challenge in pediatric patients. In such cases, the extremely small caliber of infantile bile ducts often precludes conventional end-to-side hepaticojejunostomy. While postoperative bile leakage is a known source of morbidity after pediatric liver tumor resection [1], iatrogenic injuries encountered intraoperatively require immediate and innovative reconstructive strategies. Although nonoperative management has become the standard for blunt liver trauma [2], the focus of this report is the management of acute, intraoperative hilar injury where conventional options are not feasible. Endoscopic retrograde cholangiopancreatography (ERCP) with stenting has been reported as an effective diagnostic and therapeutic modality for traumatic bile leaks in children [3]. However, in cases where the common bile duct is extremely small or injured at the hilar level, conventional end-to-side hepaticojejunostomy is often not feasible due to the high risk of anastomotic stricture.

Postoperative bile leakage is another major source of morbidity after pediatric liver tumor resection. Studies have shown that complex resections involving the hepatic hilum are associated with a higher incidence of biliary complications, and reoperation with biliary-enteric anastomosis may be required when conservative management fails [4]. In a large series of pediatric liver resections, the incidence of biliary complications such as leaks or strictures has been reported to range between 10% and 15%, particularly after extended hepatectomy [1]. Intraoperative injuries themselves are not uncommon; an analysis of more than 1,000 adult hepatectomies reported an intraoperative injury rate of 4.4%, including bile duct injuries, highlighting the importance of careful hilar dissection [5].

When hilar bile duct injuries are encountered in pediatric patients, innovative strategies are necessary. Major hepatectomy has been used in adults as a salvage procedure for complex bile duct injuries, but such an approach is not feasible in infants or young children [6]. In this context, a Kasai-like hepaticojejunostomy, utilizing a direct anastomosis of the jejunum to the hepatic plate similar to the procedure for biliary atresia, can be considered as an alternative reconstruction strategy when the bile duct is too small for standard anastomosis. Herein, we report a pediatric case of hepatoblastoma with intraoperative common bile duct injury during right hepatic trisectionectomy, managed successfully by a Kasai-type salvage reconstruction with a transanastomotic stent.

## Technical report

**Figure 1:**
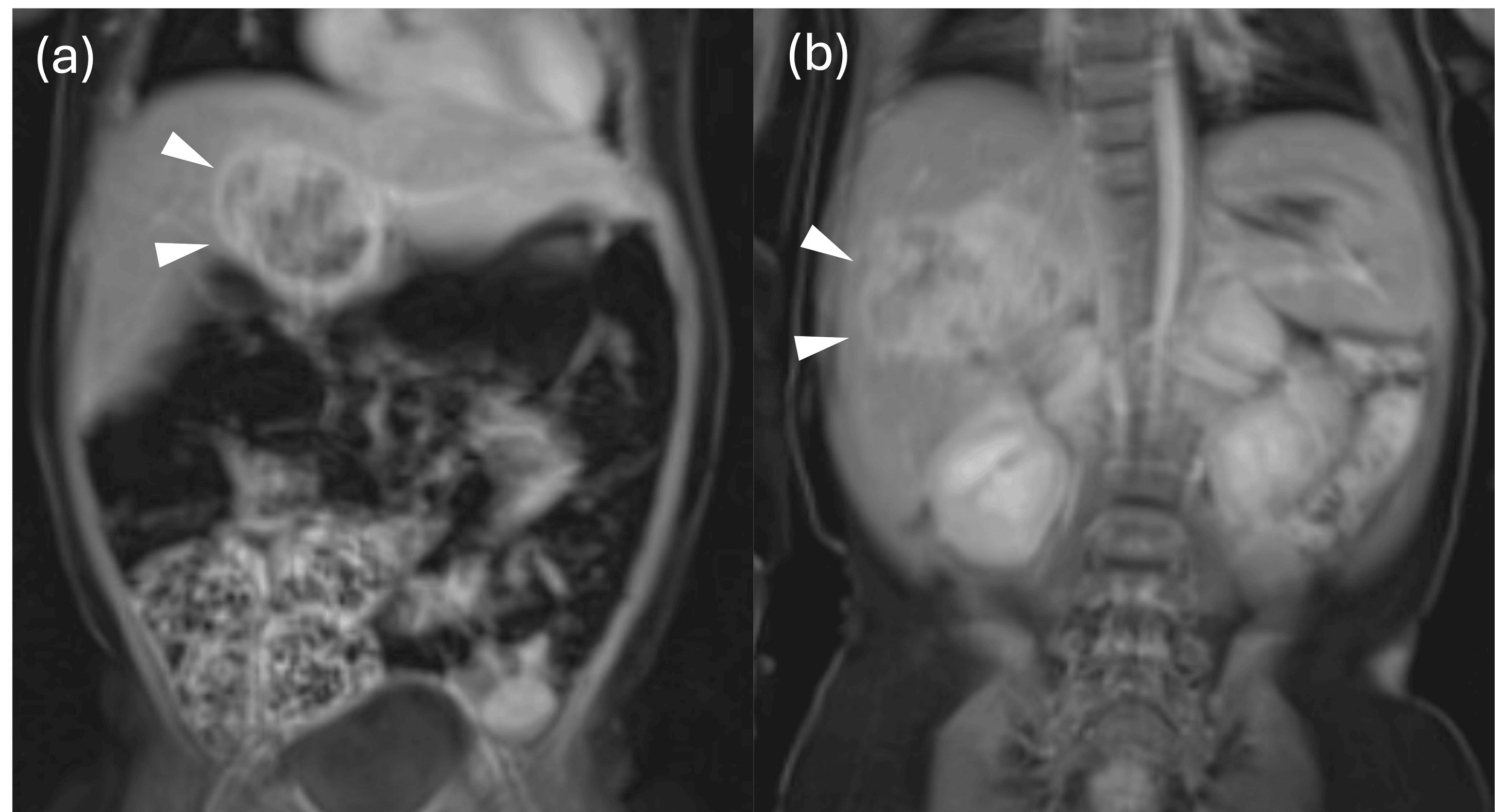
Magnetic resonance imaging (MRI) of the liver tumors (a) The segment 4 tumor and (b) the segment 6 tumor. The white arrowheads indicate the tumor.

**Figure 2:**
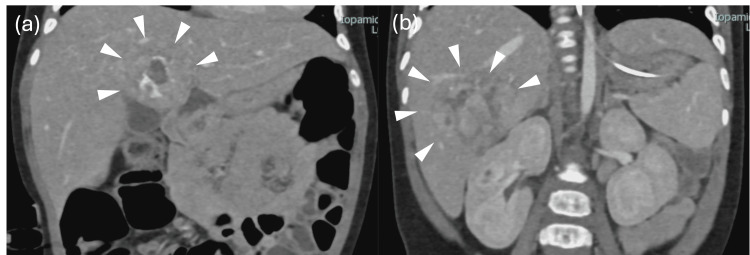
Contrast-enhanced computed tomography (CT) findings (a) The segment 4 tumor and (b) the segment 6 tumor. The white arrowheads indicate the tumor.

A one-year-old boy with multiple congenital anomalies (including craniofacial anomalies and congenital heart disease) was diagnosed with hepatoblastoma involving segments 4 and 6. After initiation of chemotherapy, progressive renal dysfunction limited further cytotoxic therapy; curative resection became the oncologic priority. Imaging demonstrated the S4 mass draping over the hepatic hilum. The relationship between the tumor and the hepatic hilum was carefully evaluated in both axial and coronal planes (Figures 1a, 1b, 2a, 2b), portending a challenging hilar exposure and potential biliary risk.

The patient underwent planned right hepatic trisectionectomy (S1 partial and S4-S8) with curative intent.

**Figure 3:**
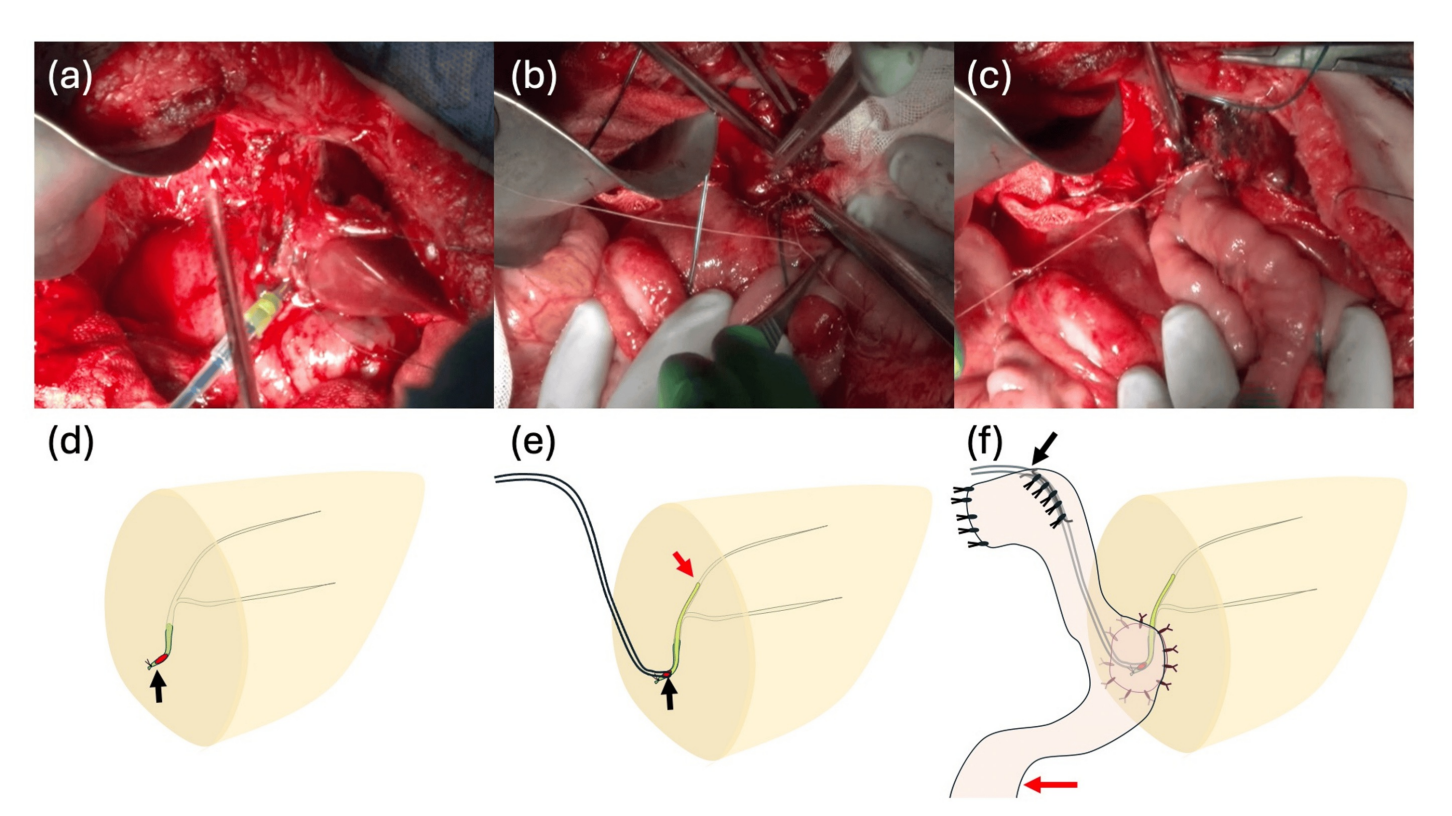
Surgical procedure and schematic illustrations of the salvage reconstruction In panels (d–f), "Proximal" indicates the orientation toward the liver, while "Distal" indicates the direction toward the distal end of the common bile duct or the aboral side (downstream) of the Roux-en-Y limb. (a, d) Extension of the bile duct injury toward the left hepatic duct to expose healthy mucosa. The black arrow (d) indicates the transected distal end of the common bile duct, and the red area indicates the site of iatrogenic injury. (b, e) Placement of a 3-Fr transanastomotic stent into the B2 duct. The stent is inserted via the injury site (black arrow, e), with its tip positioned in the B2 duct (red arrow, e). (c, f) Completed Kasai-like hepaticojejunostomy. The biliary stent is externalized through the Roux-en-Y limb (black arrow, f), which extends toward the distal (aboral) side (red arrow, f).

Through a reverse T incision, the liver appeared edematous with dilated bowel loops limiting exposure. After cholecystectomy and attempts to identify the right second-order Glissonian pedicles, the S4 tumor protruding toward the umbilical fissure obscured the portal plate. During hilar dissection, the team inadvertently dissected caudally around the common hepatic duct (CBD), resulting in a pin-hole injury. The CBD caliber measured <1 mm, an ultra-thin duct judged unsuitable for conventional end-to-side hepaticojejunostomy because of the high probability of anastomotic stenosis and technical fragility. Given the duct size and injury location, the team elected a Kasai-type salvage reconstruction. To facilitate the reconstruction, the bile duct incision was extended proximally, a "cut-up" maneuver, from the CBD toward the left hepatic duct. This maneuver was specifically performed to expose healthy, well-vascularized bile duct mucosa for the subsequent anastomosis, rather than simply to enlarge the diameter of the opening (Figure 3a, 3d). A 3-Fr ATOM tube was advanced into segment B2 as a transanastomotic stent (Figure 3b, 3e). A Roux-en-Y limb was constructed 20 cm distal to the ligament of Treitz; the limb was brought to the hepatic transection surface, and the jejunal wall was sutured to the liver plate in a hepaticojejunostomy fashion (10 interrupted 5-0 polypropylene stitches), akin to the Kasai technique (Figure 3c, 3f). The stent was externalized through the jejunal limb and fixed to the abdominal wall. Closed drains were positioned near the transection plane and the biliary reconstruction.

The child was transferred from intensive care on postoperative day (POD) 3. Early bile drainage was managed under siphon negative pressure, then transitioned to gravity. Enteral feeding was advanced stepwise. There was no bile leak clinically or radiographically. On POD 10, contrast study via the biliary drain revealed intrahepatic duct opacification without extravasation. Total parenteral nutrition was discontinued by the second postoperative week, and the patient tolerated continuous enteral feeding thereafter. The biliary stent was removed at three months postoperatively.

**Figure 4:**
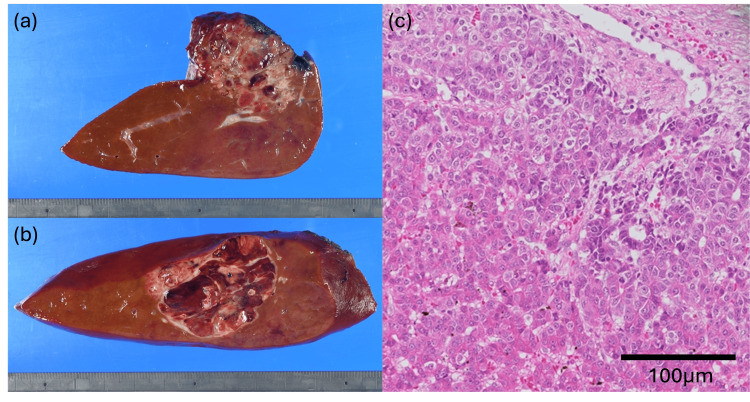
Pathological findings of the resected hepatoblastoma (a) Macroscopic appearance of the segment 4 tumor; (b) macroscopic appearance of the segment 6 tumor; (c) hematoxylin–eosin staining. Both lesions exhibited similar morphology, with atypical cells possessing enlarged nuclei and prominent nucleoli forming trabecular and solid nests, resembling the fetal subtype of hepatoblastoma. Areas of fibrosis, hemosiderin deposition, hemorrhage, calcification, coagulative necrosis, and focal osteoid formation were observed, with scattered extramedullary hematopoiesis. Histologically, the tumors were classified as post-chemotherapy hepatoblastoma, mixed epithelial and mesenchymal type, simple subtype (WHO 5th edition: mixed epithelial and mesenchymal hepatoblastoma without teratoid features). Pathological treatment effect was evidenced by approximately 40% fibrosis and necrosis. Surgical margins were free of tumor involvement.

Final pathology confirmed two intrahepatic tumors measuring 32 × 30 × 45 mm in segment 4 (Figure 4a) and 46 × 38 × 30 mm in segment 6 (Figure 4b). Both lesions exhibited similar morphology, predominantly resembling the fetal subtype of hepatoblastoma. Histologically, the tumors were classified as post-chemotherapy hepatoblastoma, mixed epithelial and mesenchymal type, simple subtype. Pathological treatment effect was evidenced by approximately 40% fibrosis and necrosis (Figure 4c). Surgical margins were free of tumor involvement.

## Discussion

Biliary complications remain a significant source of morbidity following pediatric liver resection. In a large retrospective series of 86 pediatric liver tumor resections, the incidence of biliary complications was 14%, with bile leakage being the most common [1]. Similarly, Han et al. reported six children who developed postoperative intractable bile leakage after complex liver tumor surgery, all of whom ultimately required surgical reintervention with either hepaticojejunostomy or cholecystoenterostomy [4]. These data underscore that while conservative management may suffice for minor leaks, surgical reconstruction remains indispensable for severe or persistent cases.

Endoscopic and radiologic interventions have been shown to be effective in selected children with traumatic bile duct injury or postoperative leaks [2,7]. Aljahdali et al. demonstrated the feasibility of ERCP with stent placement in three pediatric trauma cases, achieving resolution without the need for open surgery [3]. Likewise, Almaramhi and colleagues reviewed five pediatric cases of traumatic bile duct injury treated with endoscopic or radiologic intervention, all with favorable outcomes [2]. Nevertheless, in the setting of ultra-thin bile ducts, such as in neonates or infants, or when the injury is located very proximally, endoscopic management is technically impossible. In these situations, surgical alternatives must be sought.

In our case, the injured common bile duct measured less than 1 mm in diameter, precluding conventional hepaticojejunostomy. A Kasai-like hepaticojejunostomy was therefore performed, allowing mucosa-to-mucosa apposition between the hilar plate and the Roux-en-Y jejunum. Although this approach is traditionally used for biliary atresia, its adaptation in this setting enabled safe biliary drainage while avoiding the risks of anastomotic stenosis associated with fragile, tiny ducts. The patient has remained asymptomatic without any signs of cholangitis or biliary obstruction for six months after the surgery. However, the long-term prognosis of pediatric patients undergoing salvage hepaticojejunostomy for iatrogenic bile duct injury remains uncertain, and close follow-up is essential. Potential late complications, including anastomotic stricture or recurrent cholangitis, necessitate rigorous, long-term monitoring to ensure the sustained success of the reconstruction. Furthermore, while this technique offers a promising salvage option for complex hilar injuries in infants, it should be considered selectively for cases where ductal size precludes conventional anastomosis. Future studies with larger cohorts are needed to better understand the long-term implications and to refine the selection criteria for this approach. Previous studies emphasize that referral pattern and timing of repair significantly influence outcomes in bile duct injury [8]. Early recognition of biliary complications, adequate drainage, and timely surgical reconstruction are crucial to achieving favorable results. Although major hepatectomy has been advocated in adults with complex hilar bile duct injuries [6], in children, less invasive salvage strategies such as the Kasai-type approach may offer a safer and more physiologic alternative.

## Conclusions

This case demonstrates the feasibility of Kasai-like hepaticojejunostomy as a selective salvage procedure for iatrogenic hilar bile duct injury in infants. While the successful outcome in this single case is encouraging, it is important to recognize that this approach was necessitated by extreme anatomical constraints that precluded conventional reconstruction. When duct size makes standard hepaticojejunostomy technically impossible, this technique offers a safe alternative; however, further evaluation is needed to establish its long-term role compared to standard methods.
